# NEK2 Promotes Cell Proliferation and Glycolysis by Regulating PKM2 Abundance *via* Phosphorylation in Diffuse Large B-Cell Lymphoma

**DOI:** 10.3389/fonc.2021.677763

**Published:** 2021-06-08

**Authors:** Lingna Zhou, Liya Ding, Yuqi Gong, Jing Zhao, Jing Zhang, Zhengrong Mao, Zhe Wang, Wei Zhang, Ren Zhou

**Affiliations:** ^1^ Department of Pathology, First Affiliated Hospital, Zhejiang University School of Medicine, Hangzhou, China; ^2^ Cancer Institute, Department of Pathology, Second Affiliated Hospital, Zhejiang University School of Medicine, Hangzhou, China; ^3^ Institute of Clinical Science, Department of Pathology, Sir Run Run Shaw Hospital, Zhejiang University School of Medicine, Hangzhou, China; ^4^ Department of Pathology, Xijing Hospital, Fourth Military Medical University, Xi'an, China

**Keywords:** diffuse large B-cell lymphoma, GSEA, glycolysis, NEK2, PKM2

## Abstract

Diffuse large B-cell lymphoma (DLBCL) is the most frequent and commonly diagnosed subtype of NHL, which is characterized by high heterogeneity and malignancy, and most DLBCL patients are at advanced stages. The serine/threonine kinase NEK2 (NIMA-related kinase 2), a member of NIMA-related kinase (NEK) family that regulates cell cycle, is upregulated in a variety of malignancies, including diffuse large B-cell lymphoma. However, the role and underlying mechanisms of NEK2 in DLBCL have seldom been discussed. In this study, we identified that NEK2 is upregulated in DLBCL compared to normal lymphoid tissues, and overexpression of NEK2 predicted a worse prognosis of DLBCL patients. Gene set enrichment analysis indicates that NEK2 might participate in regulating glycolysis. Knockdown of NEK2 inhibited growth and glycolysis of DLBCL cells. The interaction between NEK2 and PKM2 was discovered by tandem affinity purification and then was confirmed by immunofluorescence staining, coimmunoprecipitation, and immunoprecipitation. NEK2 bounds to PKM2 and regulates PKM2 abundance *via* phosphorylation, which increases PKM2 stability. The xenograft tumor model checks the influence of NEK2 on tumor growth *in vivo*. Thus, NEK2 could be the novel biomarker and target of DLBCL, which remarkably ameliorates the diagnosis and treatment of DLBCL.

## Introduction

Non-Hodgkin lymphoma (NHL) accounts for 4% of the total number of new cancer cases recorded in 2020, ranked seventh among all cancer types (1). Diffuse large B-cell lymphoma (DLBCL) is the most frequent and commonly diagnosed subtype of NHL, which is characterized by high heterogeneity and malignancy, and most DLBCL patients are at advanced stages (2). Despite diverse sites of origin (3) and genetic heterogeneity (4), DLBCL displays a common set of hallmarks that support its continued ability to proliferate. Therefore, investigating the regulatory mechanisms promoting tumor cell growth and exploring novel biomarkers of DLBCL could remarkably ameliorate the diagnosis and treatment of DLBCL.

Due to the signaling and biological significance of kinases, there have been extensive efforts to investigate the mechanism of kinase in tumors, which contributing to developing selective kinase inhibitors with novel treatment opportunities (5). NEK2 (NIMA-related kinase 2) is a member of NIMA-related kinase (NEK) family of serine/threonine kinases that regulate cell cycle (6). NEK2 was found to be upregulated in DLBCL compared to Follicular lymphoma (FL), the latter exhibit relatively low malignancy (7). And our further differential expression analysis showed that NEK2 is upregulated in DLBCL compared to normal lymphoid tissues. As disease-specific differential expression genes reveal potential disease-related molecular mechanisms, NEK2 might play an important role in DLBCL.

Previous evidence has shown that overexpression of NEK2 in various human cancers is well correlated with processes of cancer progression, including chromosomal instability (8, 9), high proliferation, drug resistance (10), and metabolism (11). However, the role and underlying mechanisms of NEK2 in DLBCL have seldom been discussed. Our GSEA analysis indicates that NEK2 might participate in regulating glycolysis in DLBCL. Aerobic glycolysis is considered a metabolic signature of invasive cancer, which is closely associated with tumor growth and progression. Thus, novel therapeutic targets might be discovered by a better understanding of the molecular mechanisms of glycolysis in DLBCL.

In the present study, we aimed to detect the NEK2 expression in DLBCL and investigate its effect and mechanism by bioinformatic analyses as well as experimental exploration. On the basis of bioinformatic analyses, we demonstrated that NEK2 was upregulated in DLBCL compared to normal lymphoid tissue, and overexpression of NEK2 predicted a worse prognosis of DLBCL patients. It was experimentally demonstrated that NEK2 contributed to DLBCL cell proliferation *via* inducing aerobic glycolysis. Besides, we uncovered NEK2 regulates PKM2 abundance *via* phosphorylation, which is a rate-limiting glycolytic enzyme. These may contribute to the treatment of DLBCL patients.

## Materials and Methods

### Bioinformatic Analysis of NEK2 in DLBCL

To collect datasets that meet the requirements of comparative analysis between DLBCL and normal lymphoid tissue, we made a systematic search in the GEO database (**Supplementary Figure 1A**). Three datasets that depended on the GPL570 platform were collected, including GSE25638, GSE44337, and GSE56315, and their normalized gene expression profile data were downloaded by the GEOquery package. The maximum mean expression values of probes were collapsed to the genes and were annotated with the hgu133plus2.db. We extracted the expression levels of the NEK2 from the datasets. Boxplot of NEK2 mRNA expression level s for the two groups (DLBCL *vs* Normal) was drawn using R function boxplot(). Both Kaplan–Meier (KM) curves and gene set enrichment analysis (GSEA) were performed in GSE23501. Expressions of NEK2 were divided into high and low groups according to the median expression level. KM curve was plotted using survival R package. Besides, GSEA (v4.1.0) was performed to determine which pathway NEK2 might participate in. C2.cp.kegg.v6.0 symbols of the KEGG set was selected, and the number of permutations was set to 1000 for our analysis.

### Human Tumor Specimens

All the human tumor specimens, including fresh tissues and paraffin-embedded tumor sections, were collected from the Department of Pathology, the First Affiliated Hospital, Zhejiang University School of Medicine, Hangzhou, Zhejiang, China. Specimens were collected with informed consent, DLBCL was diagnosed according to the World Health Organization Classification of Tumors of Hematopoietic and Lymphoid Tissues. This study was approved by the Ethics Committee of Zhejiang University.

### Western Blotting

For sample preparation, the fresh human tissues were grinded on liquid nitrogen. While, the cell suspensions were collected separately into centrifuge tubes, and cells were pelleted by centrifugation at 800g for 3 min, followed by washing with pre-cold PBS. The samples were lysed in 1 × SDS loading buffer supplemented with protease inhibitors at 100°C for 10min. Then protein samples were fractionated by 12% SDS/PAGE and electrophoretically transferred to PVDF membranes (Millipore). After blocking in 5% non‐fat milk in TBS/Tween-20 for 2 hours, the membranes were incubated with primary antibodies against NEK2 (ab109283, Abcam), Actin (sc-47778, Santa Cruz), GAPDH (ab181602, Abcam), PKM2 (4053, Cell Signaling Technology), Phospho - (Ser/Thr) Phe (ab17464, Abcam), Flag (ab205606, Abcam), 6X His (ab213204, Abcam) at 4°C overnight. The membranes were washed three times with TBST for 10 min each time and incubated with secondary antibodies: Dylight 680-goat anti-rabbit lgG (E032720, EarthOx) or Dylight 800-goat anti-mouse lgG (E032810, EarthOx), IPKine HRP mouse anti‐rabbit IgG LCS (A25022, Abbkine) were used. Membranes with fluorescent secondary antibodies were scanned using Odyssey CLx (Li-COR), while membranes with HRP-conjugated secondary antibodies were detected with FDbio-Dura ECLkit (Fdbio Science, China) by ChemiScope 3000 chemiluminescent analyzer (CLINX, China). Protein synthesis inhibitor -Cycloheximide (CHX, J&K Scientific, 375034) was used to inhibit protein synthesis. Finally, the protein bands were quantified by the ImageJ software.

### Immunohistochemistry Staining

Slides were baked for 1 hour at 60 °C. Sections were then dewaxed using xylene I for 20 min and xylene II for 20 min, hydrated with absolute ethanol I and absolute ethanol II for 10 min, 95% ethanol, 85% ethanol, and 75% ethanol for 3min, washed three times with PBS for 5 min each. The tissue was then incubated with 3% hydrogen peroxide for 20 min at room temperature and washed three times with PBS for 5 min each. Then, the slides were boiled in EDTA antigen restore solution (ZLI-9069, Origene) by a pressure cooker to repair antigens and cooled down to room temperature. After being blocked with 10% goat serum (AR009, Boster) for 15 min at room temperature, the tissue was incubated with antibodies against NEK2 (1:100, ab115731, Abcam), Ki67 (1:600, ab92742, Abcam) at 4°C overnight, followed by for 15 min at room temperature. After washing with PBS, the slides were incubated with MaxVision Mouse/Rabbit (Kit-5020, Maixin) for 15min. Then we conducted DAB chromogenic and hematoxylin stain. Finally, after dehydration by gradient ethanol and xylene on the opposite order with dewaxing, the slides were sealed with neutral balsam. The slides were observed and scanned by an Olympus VS120 microscope. Staining index (values 0–12) was calculated by the product of the intensity of positive staining (negative, 0; weak 1; moderate, or strong, 3 scores) and the proportion of immunopositive cells (< 25%, 1; 25–50%, 2; 50–75%, 3; >75%, 4 scores).

### Quantitative Real-Time PCR

Total RNA was isolated using an RNA-Quick purification kit (RN001, ES Science) according to the manufacturer’s instructions. One µg of isolated RNA was used to synthesize cDNA by HiScript II Q RT SuperMix for qPCR (+gDNA wiper) (R233-01, Vazyme Biotech co., ltd). PCR amplification for the quantification performed using Roche LightCycler 480 SYBR Green system (04887532001, Roche). The primers used for qRT-PCR were listed in **Supplementary Table 1**.

### Cell Culture

DLBCL cell lines OCI-Ly10, Peiffer, Toledo, and Karpass422 were purchased from Shanghai EK-Bioscience Co., Ltd; SU-DHL4 was obtained from the Type Culture Collection of the Chinese Academy of Sciences; OCI-Ly3 was kindly provided by Dr. Zhe Wang from the Department of Pathology at Xijing Hospital, the Fourth Military Medical University. The DLBCL cell lines were cultured in IMDM with 10% FBS (SFBS, Bovogen). HEK293T was obtained from the Type Culture Collection of the Chinese Academy of Sciences. The culture medium of HEK293T was DMEM supplemented with 10% FBS (10270-106, Gibco). Cultures were maintained at 37°C in a humidified atmosphere containing 5% CO2.

### Construction of Vector, Lentiviral Packaging, and Cell Line Establishment

For knockdown, after digestion with BamHI and EcoRI, lentiviral vector pLVX-shRNA were recovered by gel-purification (A3612, Koning). The hairpin target sequences of non-target control (NTC) and NEK2 were listed in **Table 1**. They were subcloned into pre-cut pLVX-shRNA at EcoRI/BamHI sites. For overexpression, Full-length coding sequences were obtained from the NCBI Gene Bank. Full-length human NEK2 cDNA with a hexahistidine (6×His) tag was list in **Supplementary Table 2**. Mutagenesis information of NEK2 was obtained from UniProt/SwissProt, which identified Threonine 175 to alanine mutation and Serine 241 to alanine mutation resulted in a loss of kinase activity. Thus, an inactive His-NEK2 mutant sequence was generated by introducing T175A and S241A mutations (**Supplementary Table 3**). They were subcloned into PCDH-CMV-GFP-MCS-EF1-puro vector at EcoRI/BamHI. Flag-PKM2 were then cloned in pLVX-IRES-Neo vector at EcoRI/BamHI. The construction of the overexpression vector was performed by Youkang Biological Technology Co. Ltd, Hangzhou, China. For lentiviral packaging, psPAX2 and pMD2.G were applied as packaging and envelop plasmids, respectively. 293T cells were co-transfected with psPAX2, pMD2.G, and respective lentiviral vectors for the generation of lentivirus. Lentiviruses were concentrated by ultracentrifugation at 72,000 g for 2 hours at 4°C using Beckman XPN-100. The transduced cells were placed under the selection of 2 ng/μl puromycin for two weeks.

**Table 1:** The hairpin target sequences of non-target control (NTC) and NEK2.

Gene	Primer(5’-3’)
NTC-F	GATCCCAACAAGATGAAGAGCACCAACTCGAGTTGGTGC TCTTCATCTTGTTGTTTTTTG
NTC-R	AATTCAAAAAACAACAAGATGAAGAGCACCAACTCGAGT TGGTGCTCTTCATCTTGTTGG
shNEK2#1-F	GATCCGCAGACGAGCAAAGAAGAAATCTCGAGATTTCTTCTTTGCTCGTCTGCTTTTTTG
shNEK2#1-R	AATTCAAAAAAGCAGACGAGCAAAGAAGAAATCTCGAGATTTCTTCTTTGCTCGTCTGCG
shNEK2#2-F	GATCCGAGGAAGAGTGATGGCAAGATCTCGAGATCTTGCCATCACTCTTCCTCTTTTTTG
shNEK2#2-R	AATTCAAAAAAGAGGAAGAGTGATGGCAAGATCTCGAGATCTTGCCATCACTCTTCCTCG

F, forward; R, reverse.

### Proliferation Assay

Cell Counting Kit-8 (CCK8) assay and 5-ethyn yl-2′-deoxyuridine (EdU) staining were used for assessing proliferation. For the CCK8 assay, the cells were resuspended in Fresh IMDM containing 10% FBS and counted by a cell counting plate. Cells (1 × 10^4^/well) were cultured in a 96-well plate, and medium without inoculation was used as the blank control. They received the CCK8 detection at the given time (0h, 24h, 48h, 72h). Ten μL CCK8 reagent was added to the culture medium two hours before analysis. Optical density (OD) 450 values were measured using Spectra Max M5. Cell viability fold change was calculated by the following formula: Cell viability fold change = (OD_t_ − OD_tblank_)/(OD_0h_ − OD_0hblank_). For Edu staining, the cells were resuspended with Edu (10 μmol/L) in Fresh IMDM containing 10% FBS and counted by a cell counting plate. Cells (5 × 10^5^/well) were cultured in a 24-well plate for 2 hours. Then we followed standard staining procedures (C10310-1, RiboBio). After performing 4′,6‐diamidino‐2‐phenylindole (DAPI) staining, we sealed the sections with an anti-fluorescence quencher. Olympus FV1000 laser scanning confocal microscopy (Olympus, Japan) was used to obtain images. The percentage of EdU-positive cells calculated by the following formula: EdU-positive rate = EdU-positive cell count/(EdU-positive cell count + EdU-negative cell count) × 100%.

### Measurement of Glucose Consumption and Lactate Secretion

The cells were resuspended in Fresh IMDM containing 10% FBS and counted by a cell counting plate. Cells (5 × 10^5^/well) were seeded into a 6-well plate, and medium without inoculation was used as the control. After cultured for 24h, the culture medium was collected to measure glucose and lactate concentrations. The glucose concentrations were assayed using the Glucose Assay Kit (E1010, Applygen Technologies Inc.) based on the glucose oxidase method. Optical density (OD) 550 values were measured using Spectra Max M5. Glucose concentration (mmol/L) = concentration standard (mmol/L) × (OD_sample_- OD_blank_)/(OD_standard product_- OD_blank_). Glucose consumption was calculated by deducting the measured glucose concentration in the medium of the experimental groups from the glucose concentration of the control. Lactate production was determined by using the Lactate Assay Kit (A019-2-1; Nanjing Jiancheng Bioengineering Institute) according to the manufacturer’s instructions. Optical density (OD) 530 values were measured using Spectra Max M5. Lactate concentration (mmol/L) = concentration standard (mmol/L) × (OD_sample_- OD_blank_)/(OD_standard product_- OD_blank_). PKM2 inhibitor (HY-103617, Medchemexpress).

### Measurement of Extracellular Acidification Rate (ECAR)

Cells were seeded at 100,000 cells per well in a 96-well cell culture XF microplate (Seahorse Biosciences) coated with poly-d lysine overnight. Cells were incubated with XF base medium at 37°C without CO2 for one hour. Final concentrations of 10 mM glucose, 1 μM oligomycin and 50 mM 2-deoxyglucose (2-DG) were used and were sequentially injected at the indicated time points. Data were analyzed with software: Wave 2.3.

### Immunofluorescence Staining

The cell suspensions were collected separately into centrifuge tubes, and cells were pelleted by centrifugation at 800g for 3 min. Then cells were washed twice in PBS, smeared onto glass slides, air-dried, and fixed in 3.7% formaldehyde at 37°C for 15 min, followed by 0.1% Triton X-100 treatment at room temperature for 10 min. Then the slides were blocked with Goat serum for 1 h at room temperature. After that, cells were incubated with the antibody against NEK2 (1:50, sc-55601, Santa Cruz) and PKM2 (1:100, 4053, Cell Signaling Technology) overnight at 4°C. Then cells were incubated with Alexa Fluor 488 goat anti-rabbit lgG (A11008, Life Technologies) and Alexa Fluor 594 goat anti-mouse lgG (A11005, Life Technologies) for 1h at room temperature and washed with PBS. Nuclei were stained with 4’,6-diamidino-2-phenylindole (DAPI) for 5 min, protected from light. Finally, the sections were sealed by an anti-fluorescence quencher (AR1109, Boster). Olympus FV1000 laser scanning confocal microscopy (Olympus, Japan) was used to obtain images.

### Immunoprecipitation (IP)

Cells were collected and lysed in cell lysis buffer for western blot and IP (P0013, Beyotime) for 1h on ice. Lysates were then centrifuged at 15,000 × g for 10 min at 4°C. A fraction of supernatant was removed as input, and the remaining supernatants were mixed with the primary antibody and IgG for the negative control, respectively. Then they were incubated in a rotary shaker for 6 h at 4°C. Then, protein A/G PLUS agarose (sc-2003, Santa Cruz) was added to the mixture and incubated in a rotary shaker at 4°C overnight. The beads were collected by centrifuging at 5000g for 3 min at 4°C and washed 2 times with 200 μl of ice-cold PBS. 1X SDS was mixed with the beads and boiled for 10 min, followed by western blot analysis. The following antibodies, including NEK2 (sc-55601, Santa Cruz), PKM2 (4053, Cell Signaling Technology) and normal mouse lgG (sc-2025, Santa Cruz), were used in the IP assays.

### Co-Immunoprecipitation (CO-IP) Assay

293T cells (5 × 10^5^/well) were cultured in a 6-well plate and cultivated overnight. Then the cells were adherent and could be used for transfection. 1ug plasmids and 10μl Lipofectamine 2000 reagent (P/N 52887, Invitrogen) were dissolved in 250μl of OPTI-MEM medium (31985-070, Gibco) respectively and stand at room temperature for 5min. The mixtures were then combined and left at room temperature for 20 minutes. The mixture was added to the plate and incubated for 6-8h. After transfection, the medium was replaced with fresh DMEM containing 10% FBS. Cells were harvested for 48 hours and used to perform CO-IP. The following steps were the same as IP. Anti-Flag M2 magnetic beads (M8823, Sigma) and 6X His (ab213204, Abcam) were used in CO-IP assay.

### *In Vivo* Experiments

Cells were collected and counted by a cell counting plate. Then cells were pelleted by centrifugation at 800g for 3 min. 10^6^cells were resuspended by 50μl PBS and 50μl Matrigel (354234, Corning). Four NOD-SCID mice (six-week-old) were injected into the right flank with 10^6^ non-target control OCI-Ly3 cells and into the left flank with 10^6^ NEK2 knockdown OCI-Ly3 cells. We measured the tumor size every 2 days using digital calipers in two dimensions until the right tumor volumes reached an average of 50-100 mm^3^. Tumor volumes were calculated using the following formula: V = length × (width)^2^/2. Mice were euthanized with carbon dioxide when the tumor size reached 1000mm^3^. The xenograft was quickly taken out and weighed.

### Statistical Analysis

GraphPad Prism software8.0.0 was used to perform Statistical analysis. Differences between groups were compared by an independent-sample t-test. P values <0.05 were considered significant.

## Results

### NEK2 Is Highly Expressed in DLBCL and Indicate a Poor Prognosis

**Figure 1:**
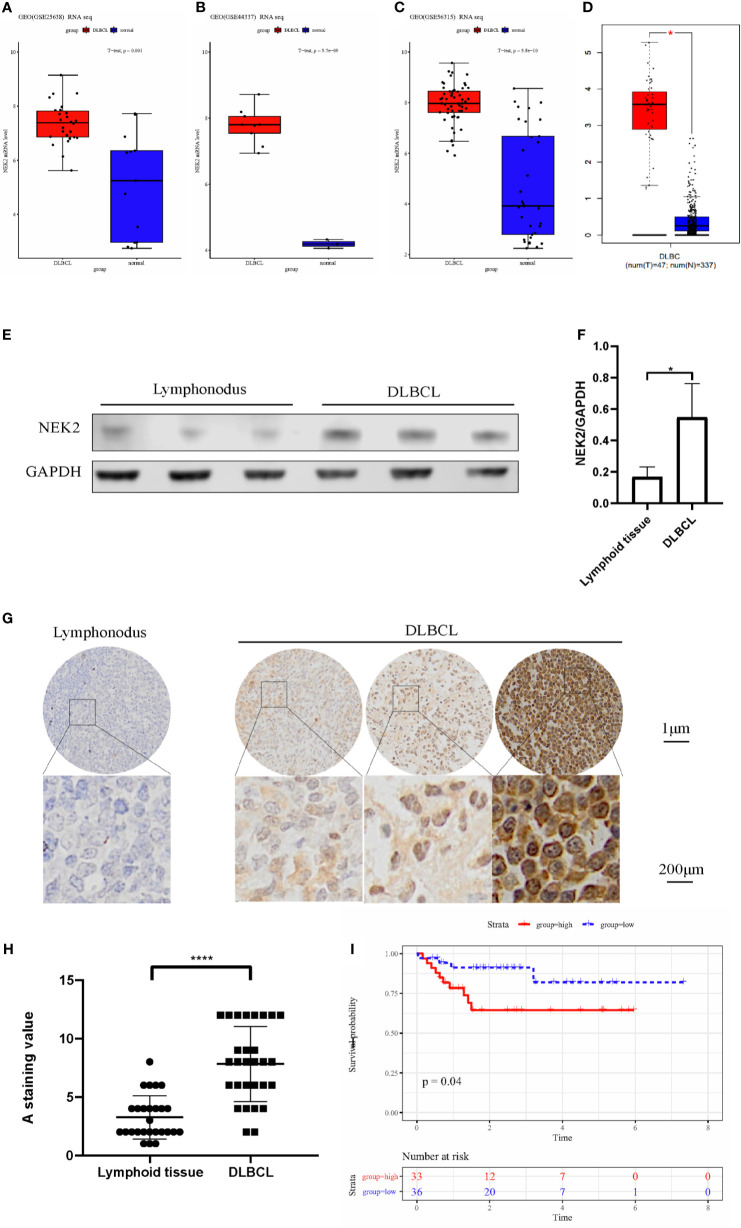
NEK2 is highly expressed in DLBCL and indicates a poor prognosis. **(A–D)** Box plots for the expression level of NEK2 in GSE25638, GSE44337, GSE56315, and GEPIA; **(E)** NEK2 protein expression level of three lymph node samples and three patients with DLBCL; **(F)** Bar graph of NEK2/GAPDH relative ratio in lymph node samples and patients with DLBCL (*P < 0.05, Student’s t-test); **(G)**The expression of NEK2 was characterized by immunohistochemistry (IHC) analysis. IHC was performed using formalin-fixed paraffin-embedded samples from 26 lymphoid tissues and 28 DLBCL patients. Staining intensity was graded as presented: negative, weak, moderate, and strong; **(H)** Grouped scatter plots of IHC score of lymphoid tissues and DLBCL specimen (****P < 0.0001, Student’s t-test); **(I)** Kaplan-Meier survival curves by survival analysis of NEK2 in GSE23501.

After screening, three datasets were used to evaluate the expression level of NEK2 in DLBCL, which clinical information is summarized in the **Supplementary Table 4**. According to our analysis, NEK2 was highly expressed in DLBCL compared with normal lymphoid tissues (**Figures 1A–C**). Furthermore, the result obtained from the Gene Expression Profiling Interactive Analysis (GEPIA) database was consistent with our analysis (**Figure 1D**). To confirm the differences in expression at the protein level, western blot (WB) and Immunohistochemical (IHC) analyses of NEK2 in DLBCL samples and normal controls were performed. 6 specimens, including 3 DLBCL patient tumor samples and 3 non-cancer patient lymphoid tissue samples, were subjected to WB assay. As is shown in **Figures 1E, F**, the result of WB revealed a significantly higher expression of NEK2 in DLBCLs compared to lymphoid tissue. Besides, IHC was conducted using formalin-fixed paraffin-embedded samples from 28 DLBCL patient tumor samples and 26 non-cancer patient lymphoid tissues. Staining intensity was graded as presented: negative, weak, moderate, and strong, which were shown in **Figure 1G**. The immunoreactivity score of immunohistochemistry also confirmed the elevation of NEK2 in tumor samples (**Figure 1H**). Kaplan-Meier survival analysis was used to determine survival with respect to NEK2 in dataset GSE23501. Increased expression of NEK2 predicted a significantly shorter overall survival (**Figure 1I**). These data suggested that NEK2 is highly expressed in DLBCL and indicates a poor prognosis. Therefore, we speculated that NEK2 might play an important role in DLBCL.

### NEK2 Promotes Cell Proliferation

**Figure 2:**
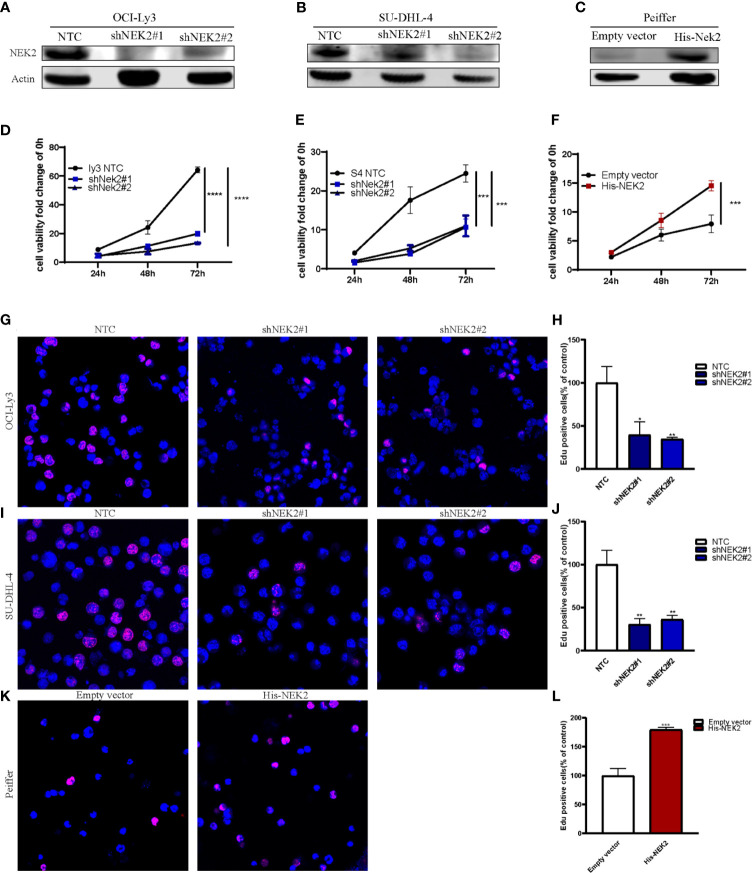
NEK2 promotes cell proliferation and inhibits apoptosis. **(A, B)** Two different shNEK2 sequences were used (#1 and #2). Western blotting confirmed the NEK2 was efficiently knocked down in OCI-Ly3 and SU-DHL-4 cells; **(C)** Peiffer was infected by NEK2-overexpressing lentivirus. Western blotting showed successful over-expression of NEK2 in Peiffer cells; **(D–F)** NEK2 increased cell viability as reflected by CCK-8 proliferation assay (****P < 0.0001, ***P < 0.001, Student’s t-test); **(G–L)** Representative images of EdU staining and histogram statistics of Edu-positive cell rate, which also reflected NEK2 increased cell proliferation (*P < 0.05, **P < 0.01, ***P < 0.001, Student’s t-test).

Abnormal proliferation is characteristic of Malignant tumors, and *in vitro* proliferation assay further validated the role of NEK2 in the proliferation of DLBCL cells. To select DLBCL cell lines for further investigations, we analyzed the expression status of NEK2 in DLBCL cell lines, and our results demonstrated that NEK2 expression was higher in OCI-Ly3 and SU-DHL-4 cells and lower in Peiffer cells (**Supplementary Figure 2**). Next, we generated stable shRNA expression in DLBCL cell lines of OCI-Ly3 and SU-DHL-4. At the same time, stable overexpressing cells were constructed in Peiffer cells. The efficacy of knockdown was validated by quantitative RT-PCR and WB with NEK2 antibodies (**Figures 2A–C**). To confirm the role of NEK2 in proliferation, we performed CCK-8 proliferation assay and Edu staining. Decreased NEK2 expression inhibited the viability of OCI-Ly3 and SU-DHL-4 cells (**Figures 2D, E**). Instead, overexpression of NEK2 results in the opposite direction (**Figure 2F**). We also estimated the level of proliferation rate by determining the ratio of EdU-positive cells. As shown in **Figures 2G–L**, EdU-positive cells of NEK2 knockdown groups in OCI-Ly3 and SU-DHL-4 presented in the regions with a lower cell density compared with control group. Also, EdU-positive cells of NEK2 overexpression group in Peiffer presented in the regions with a higher cell density compared with the control group. The results of the two methods were consistent. Thus, NEK2 played a positive role in the growth of DLBCL.

### NEK2 Promoted Glycolysis in DLBCL Cells

**Figure 3:**
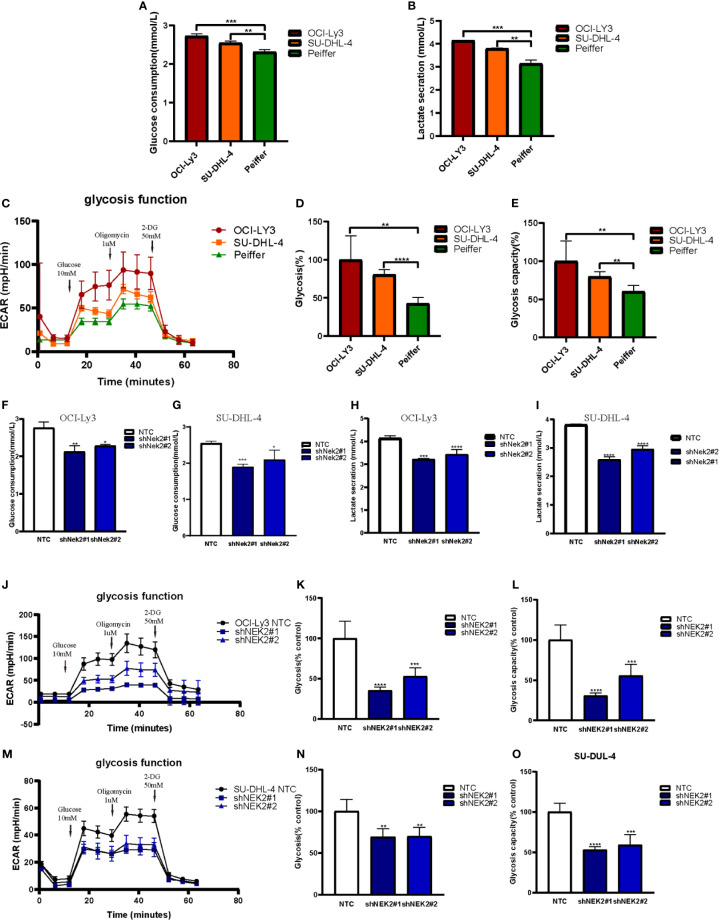
NEK2 promoted aerobic glycolysis in DLBCL cells. **(A, B)** Glucose uptake and lactate production were analyzed in OCI-Ly3, SU-DHL-4 cells, and Peiffer cells cultured at normoxia (**P < 0.01, ***P < 0.001, Student’s t-test); **(C)** Representative image of ECAR measurement in OCI-Ly3, SU-DHL-4 cells, and Peiffer cells; **(D–E)** OCI-Ly3 and SU-DHL-4 had higher glycolytic rate and more robust glycolytic capacity compared with Peiffer (**P < 0.01, ****P < 0.0001, Student’s t-test); **(F–I)** Glucose uptake and lactate production were analyzed in NEK2 silencing and control cells of OCI-Ly3 and SU-DHL-4 cultured at normoxia; (*P < 0.05, **P < 0.01, ***P < 0.001, ****P < 0.0001, Student’s t-test) **(J, M)** Representative image of ECAR measurement in NEK2 silencing and control cells of OCI-Ly3 and SU-DHL-4; **(K, L, N, O)** Knockdown of NEK2 decreased glycolysis and glycolytic capacity of DLBCL cells (**P < 0.01, ***P < 0.001, ****P < 0.0001, Student’s t-test).

With the aim of identifying the potential biological function of NEK2 in DLBCL, GSEA was performed to search the enriched KEGG pathways in GSE23501. Several pathways related to glucose energy metabolism were enriched in the NEK2 high expression group (**Supplementary Figure 3**). In addition, we compared the glycolytic function in DLBCL cell lines characterized by high *vs*. low expression of NEK2 (OCI-Ly3/SU-DHL-4 *vs* Peiffer cells) through three assays, including glucose uptake, lactate production and ECAR. The cellular glycolytic activity was determined by glucose uptake and lactate production. As shown in **Figures 3A, B**, OCI-Ly3 and SU-DHL-4 had stronger glycolytic activity compared with Peiffer. Glycolysis rate and glycolytic capacity can be ascertained by the ECAR data. We observed that OCI-Ly3 and SU-DHL-4 had higher glycolytic rate and more robust glycolytic capacity compared with Peiffer (**Figures 3C–E**). Thus, we supposed that NEK2 participated in the regulation of aerobic glycolysis. Further experiments confirmed that knockdown of NEK2 impaired glucose uptake and lactate production (**Figures 3F–I**). ECAR data also showed that knockdown of NEK2 decreased glycolysis and glycolytic capacity, reflecting the positive role of NEK2 in glycolysis of DLBCL cells (**Figures 3J–O**). Therefore, NEK2 was identified to promote glycolysis in DLBCL cells.

### NEK2 Bound to PKM2 and Increased the Phosphorylation Level of PKM2 Protein in DLBCL Cells

**Figure 4:**
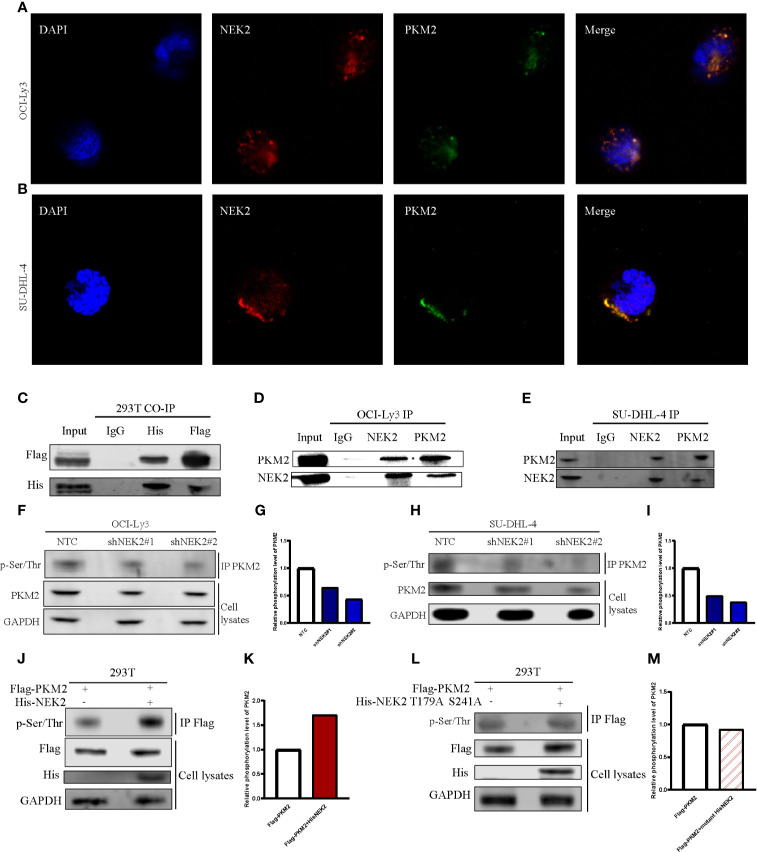
NEK2 bound to PKM2 an increases PKM2 phosphorylation in DLBCL cells. **(A, B)** The immunofluorescent staining of nuclei (blue), NEK2 (red) and PKM2 (green) in OCI-Ly3 and SU-DHL-4 cells; **(C)** The interplay between NEK2 and PKM2 was successfully detected by Co-IP; **(D, E)** The endogenous interaction between NEK2 and PKM2 proteins in OCI-Ly3 and SU-DHL-4 cells was analyzed by IP; **(F–I)** After infection with Non-target control (NTC) shRNA or NEK2 shRNA lentiviral vector, PKM2 Ser/Thr phosphorylation in OCI-Ly3 and SU-DHL-4 cells were examined by representative IP experiments and quantification of relative PKM2 phosphorylation level were shown in box plots. Knockdown of NEK2 decreased PKM2 Ser/Thr phosphorylation in SU-DHL-4 cells; **(J, K)** 293T cells were co-transfected with His-tagged NEK2 vector or control vector as well as Flag-tagged PKM2 vector. PKM2 Ser/Thr phosphorylation was examined by representative IP experiments, and quantification of relative PKM2 phosphorylation level was shown in a box plot. Phosphorylation of Flag-tagged PKM2 was increased by forced expression of His-tagged NEK2 in 293T cell; **(L, M)** 293T cells were co-transfected with empty vector or His-tagged NEK2 T179A S241A mutant (Threonine179 to alanine mutation and Serine241 to alanine mutation), along with Flag-tagged PKM2. Inactivated NEK2 failed to increase the phosphorylation of PKM2.

NEK2 has been identified as a pro-oncogenic protein that facilitates the growth and glycolysis of tumor cells. To identify NEK2 interacting proteins in DLBCL cells, we performed a TAP-MS analysis. For the reduction of nonspecific binding, we transfected plasmid containing His-tagged NEK2-cDNA into a DLBCL cell line OCI-Ly3 by lentiviral delivery. The pulled-down proteins were analyzed on mass spectrometry using sequential His antibodies immunoprecipitation. The TAP-MS analysis showed that NEK2 binds at least 50 proteins (data not shown). We specifically focused on PKM2 as it is a key enzyme in the regulation of glycolysis. Then we conducted immunofluorescence, Co-IP, and IP experiments to further confirm the interaction between NEK2 and PKM2. Immunofluorescence images of OCI-Ly3 and SU-DHL-4 showed that NEK2 is co-localized with PKM2 in the cytoplasm (**Figures 4A, B**). 293T cells were transfected with His-NEK2 and Flag-PKM2 plasmids for Co-IP. As shown in **Figures 4C**, the interplay between NEK2 and PKM2 was successfully detected by Co-IP. In addition, endogenous IP also showed that NEK2 could bind to PKM2 in OCI-Ly3 and SU-DHL-4 cells (**Figures 4D, E**). To investigate if NEK2 regulates the phosphorylation level of PKM2 protein the phosphorylation level of PKM2 protein as a kinase, we detected the threonine/serine phosphorylation of PKM2 in control group and NEK2 knockdown group of OCI-Ly3 and SU-DHL-4 cells. The results showed that the knockdown of NEK2 decreased the threonine/serine phosphorylation of PKM2 (**Figures 4F–I**). In addition, 293T cells were co-transfected with His-tagged NEK2 vector or control vector as well as Flag-tagged PKM2 vector. Indeed, the transfection of His-tagged NEK2 resulted in increased PKM2 phosphorylation on threonine/serine residues compared to the control vector (**Figures 4J, K**). Moreover, when NEK2 was inactivated by mutation T175A and S241A, it couldn’t adjust the phosphorylation level of PKM2 (**Figures 4L, M**). Therefore, we identified that NEK2 bound to PKM2 and increased the phosphorylation level of PKM2 protein in DLBCL cells.

### NEK2 Increased the Abundance of PKM2 Protein by Enhancing PKM2 Stability *via* Inducing the Phosphorylation

**Figure 5:**
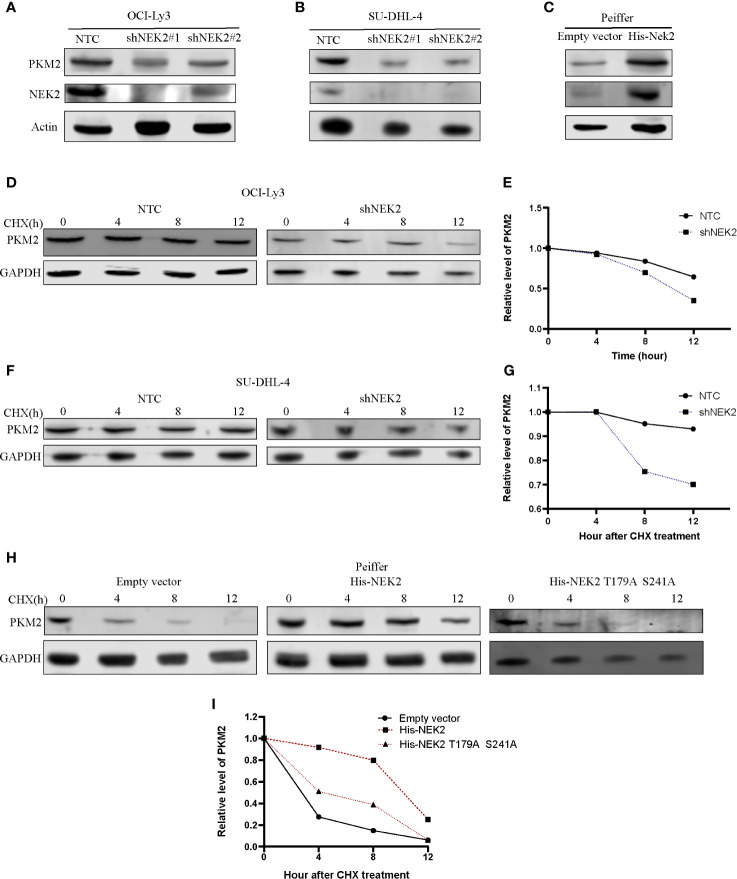
NEK2 increased the abundance of PKM2 protein and regulated the stability of the PKM2 protein in DLBCL cells. **(A, B)** Knockdown of NEK2 decreased PKM2 protein in OCI-Ly3 and SU-DHL-4 cells; **(C)** PKM2 protein abundance was increased by forced expression of NEK2 in Peiffer cells; **(D–G)** Cycloheximide (CHX) treatment was conducted to monitor the protein half-life of PKM2 in OCI-Ly3 and SU-DHL-4 cells. The PKM2 turnover rate in OCI-Ly3 and SU-DHL-4 cells with NEK2 knockdown was lower than control; **(H, I)** Cycloheximide (CHX) treatment was conducted to monitor the protein half-life of PKM2 in Peiffer cells, overexpression of NEK2 increased the half-life of the PKM2 while inactivated NEK2 (His- NEK2 T179A S241A) failed to increase the half time of PKM2.

Post-translational modifications, including phosphorylation, are directly important for the protein stability and functional activity of PKM2. We next examined whether the interaction between NEK2 and PKM2 was able to influence PKM2 abundance in DLBCL cells. For this purpose, we transfected two different NEK2 shRNAs into OCI-Ly3 and SU-DHL-4 cells, which effectively reduced NEK2 level in OCI-Ly3 and SU-DHL-4 cells and subsequently resulted in the decreased level of PKM2 protein (**Figures 5A, B**). While overexpression of NEK2 in Peiffer cells led to the increased level of PKM2 protein (**Figure 5C**). We then hypothesized that NEK2 increased PKM2 level in DLBCL cells by affecting its protein stability. To test the protein stability, CHX chase assays were used to evaluate the protein stability of PKM2. As shown in **Figures 5D–G**, the half-life of PKM2 protein decreased substantially with decreasing level of NEK2 in OCI-Ly3 and SU-DHL-4 cells. Instead, overexpression of NEK2 in Peiffer cells remarkably increased the half time of PKM2 protein. However, the inactivated NEK2 couldn’t increase the stability of PKM2 (**Figures 5H, I**). Thus, we concluded that NEK2 increased the abundance of PKM2 protein by enhancing PKM2 stability *via* inducing phosphorylation.

### NEK2 Enhanced the Proliferation and Glycolysis of DLBCL Cells Through PKM2

**Figure 6:**
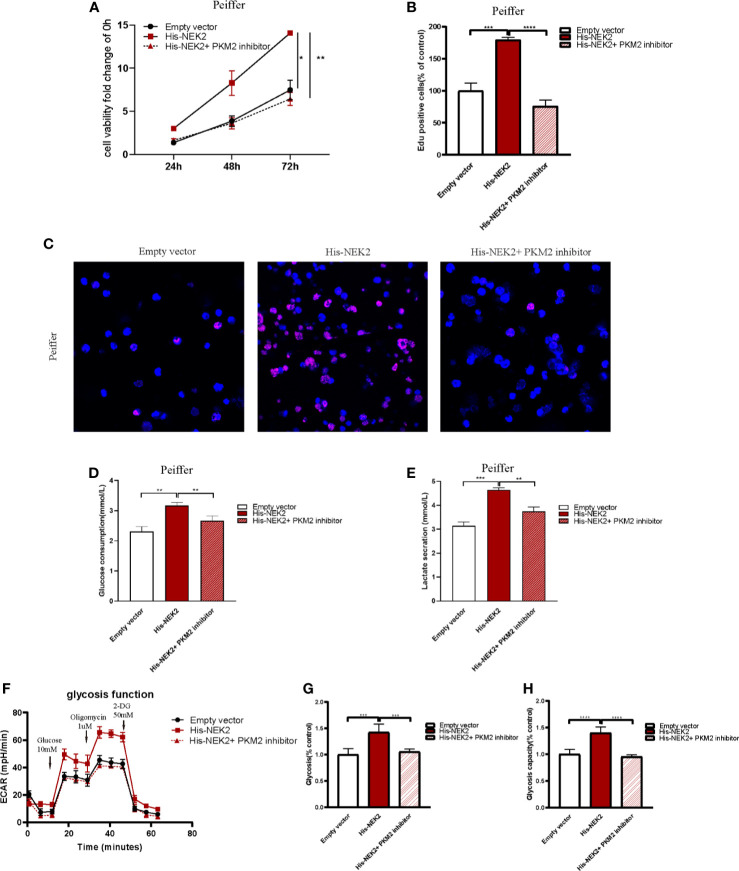
NEK2 enhanced the proliferation and glycolysis of DLBCL cells through PKM2. **(A)** Cell proliferation curves plotted according to CCK8 assay in three groups, including control group, NEK2 overexpression group and NEK2 overexpression with PKM2 inhibitor group (*P < 0.05, **P < 0.01, Student’s t-test); **(B, C)** histogram statistics of Edu-positive cell rate and representative images of EdU staining in three groups, including control group, NEK2 overexpression group and NEK2 overexpression with PKM2 inhibitor group (***P < 0.001, ****P < 0.0001, Student’s t-test); **(D, E)** Overexpression of NEK2 significantly increased the glucose uptake and lactate production of Peiffer cells while PKM2 inhibitor abrogated the promoting effects of NEK2 on the glycolysis of Peiffer cells (**P < 0.01, ***P < 0.001, Student’s t-test). **(F–H)** ECAR results in three groups, including control group,group, NEK2 overexpression group and NEK2 overexpression with PKM2 inhibitor group. Overexpression of NEK2 significantly increased the glycolytic rate and glycolytic capacity of Peiffer cells while PKM2 inhibitor abrogated the promoting effects of NEK2 on the glycolysis of Peiffer cells (***P < 0.001, ****P < 0.0001, Student’s t-test).

Next, we studied whether NEK2 modulated the growth and glycolysis of DLBCL cells through PKM2. Cell proliferation curve measured with CCK-8 showed that cell viability of Peiffer with NEK2 overexpression was significantly higher than the control group and NEK2 overexpression with PKM2 inhibitor treatment group (**Figure 6A**). Consistently, EdU staining also revealed that the proliferation rate of Peiffer with NEK2 overexpression was significantly higher than the control group and NEK2 overexpression with PKM2 inhibitor treatment group (**Figures 6B, C**). Thus, both two proliferation assays showed that overexpression of NEK2 boosted the growth of DLBCL cells, and PKM2 inhibitor significantly decreased the growth-promoting effect of NEK2. Likewise, we transfected the NEK2 vector into Peiffer cells which significantly increased glucose consumption, lactate production, glycolysis rate, and glycolytic capacity. While these promoting effects on cell glycolysis were abrogated by PKM2 inhibitor (P < 0.05, **Figures 6D–H**). The results illustrated that NEK2 enhanced the proliferation and glycolysis of DLBCL cells through PKM2.

### NEK2 Facilitates Tumor Growth of DLBCL Cells *In Vivo*

**Figure 7:**
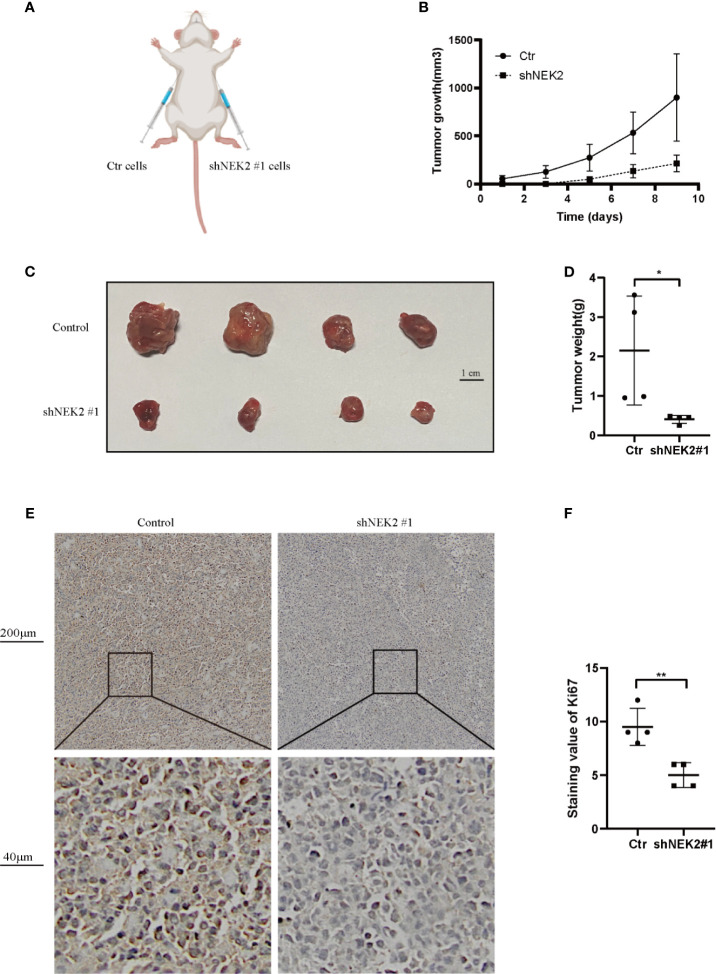
NEK2 promotes tumorigenicity *in vivo*. **(A)** Scheme of xenograft studies with shNEK2- or non-target control shRNA-expressing cells; **(B)** Growth curves of xenografted tumors which were monitored every 2 day; **(C)** Photograph of xenograft tumors. **(D)** Bar chart represents tumor weight of xenografted tumors, knockdown of NEK2 significantly decreased the growth of DLBCL cells in NOD/SCID mice (*P < 0.05, Student’s t-test); **(E, F)** The tumor sections were subjected to IHC staining with ki-67, the IHC score of Ki67 of the control group was significantly higher than knockdown of NEK2 group (**P < 0.01, Student’s t-test).

To further verify our findings *in vitro*, *in vivo* experiments were carried out. As shown in **Figure 7A**, to establish xenograft tumor model in NOD/SCID mice, OCI-Ly3 cells were transduced with the non-target control (NTC) or NEK2 shRNA and then implanted in mice. Knockdown of NEK2 significantly decreased the growth of OCI-Ly3 cells in NOD/SCID mice (**Figure 7B**). When the tumor size reached 1000mm^3^, the mice were killed, and the xenograft tumors were removed from the mice models (**Figure 7C**). Then xenograft tumors were weighted. As **Figure 7D** shows, final xenograft tumor weights in the control group were significantly higher than that in the NEK2 knockdown group. Ki67 expression is closely related to the growth of tumor cells and is one of the most frequent proliferation markers for tumors. So, we further assessed the proliferation of tumor by Ki67 staining, and the results showed Ki67 staining scores in the control group were significantly higher than NEK2 knockdown group. (**Figures 7E, F**). These data demonstrate that NEK2 promotes the growth of DLBCL cells *in vivo*.

## Discussion

Prior studies have noted the pivotal cancer-promoting function of NEK2 in cancer, which has different mechanisms among different cancer types. Several reports have demonstrated the role of NEK2 in B Lineage Commitment and Development and B cell malignancies. Gu Z et al. discovered that NEK2 contributed to the spontaneous formation of germinal center and enhanced T cell-dependent immune response (12). As the most common non-Hodgkin lymphoma in adults, the malignancy of FL was relatively low compared to DLBCL. The rate of transformation from the FL to a high-grade lymphoma in 10 years is 31%, among which DLBCL was the most frequent transformation type. De Vo S discovered that levels of mRNA transcription and protein expression of NEK2 substantially increased in the DLBCL compared with FL. Thus, the author speculated that abnormally high expression of NEK2 might drive lymphoma progression. Besides combining bioinformatics analysis and verification in clinical samples, we firstly identified that NEK2 is significantly upregulated in DLBCL compared with normal lymphoid tissue. Furthermore, Kaplan-Meier survival analysis indicated that increased expression of NEK2 predicted a significantly shorter overall survival. These data reflect the import role of NEK2 in DLBCL. However, no studies investigate the effect and mechanism of NEK2 in DLBCL.

To cover the research gap, we knocked down and overexpressed NEK2 in DLBCL cell lines to determine if NEK2 has effects on DLBCL. The experimental data showed that NEK2 promotes growth and glycolysis of DLBCL, which consistent with our GSEA result. Besides, glycolytic function in DLBCL cell lines characterized by high NEK2 expression (OCI-Ly3 and SU-DHL-4) was stronger than Peiffer characterized by low NEK2 expression. Elevated glycolysis, a predominant characteristic of Cancer cell metabolism, is highly dependent on dysregulated enzymes in metabolic processes, which could be utilized in targeted cancer therapy (13).

Here, we discovered that PKM2 was a novel binding partner for NEK2 by tandem affinity purification. The interaction of NEK2 and PKM2 was also identified by immunofluorescence, CO-IP, and IP. Pyruvate kinase (PK), a rate-limiting enzyme of glycolysis, controls the final step in glycolysis by irreversibly converting phosphoenolpyruvate (PEP) to pyruvate, which is accompanied by the production of ATP (14). PKM2 is one of four isomers of Pyruvate Kinase in mammals and ubiquitously expressed in rapidly proliferating cells, especially tumor cells and embryos (15, 16). It has been well documented that PKM2 functions as a kinase to participate in metabolic reprogramming as well as a transcriptional co-activator to regulate gene expression, which was highly related to cell growth and metastasis of tumors (17–19). Furthermore, it has been demonstrated that the expression and functions of PKM2 are regulated by post-translational modifications, including phosphorylation. Q Xu et al. (20) revealed that HSP90 promotes cell glycolysis, proliferation and inhibits apoptosis by regulating PKM2 abundance *via* Thr-328 phosphorylation in hepatocellular carcinoma. Iansante V et al. (21) reported that JNK1 increases PKM2 activity by phosphorylating PKM2 at Thr365 in hepatocellular carcinoma.

Gu et al.’s (11) research discovered that NEK2 interacts with hnRNPA1/2 in the nucleus of myeloma cells, which enhances hnRNPA1/2 binding to the intronic sequences flanking exon 9 of PKM pre-mRNA and then increase the ratio of PKM2/PKM1. However, the relationship between NEK2 and PKM2/PKM1 in DLBCL of our study is not identical to Gu et al.’s study (**Supplementary Figure 5**). Due to the complexity of the molecular regulation mechanism in tumor pathogenesis, crosstalk between NEK2 and PKM2 may be caused by different molecular mechanisms in different kinds of tumors. In the present study, we verified that NEK2 interacts with PKM2 directly in the cytoplasm of DLBCL. We then examined whether NEK2 regulated the level of phosphorylation of PKM2 as a kinase. This perception was supported by the experimental data of the present study, which shows NEK2 could increase the PKM2 phosphorylation, and the upregulation was eliminated when NEK2 was inactivated by mutation T175A and S241A. Besides, we discovered that knockdown of NEK2 reduced PKM2 level while overexpression of NEK2 increased PKM2 level in DLBCL cell. As we know, post-translational protein modifications play important roles in regulating the expression and function of PKM2. Our CHX treatment further verified that NEK2 regulated the phosphorylation of PKM2 by direct interaction, which could affect PKM2 stability. Scansite (22), an online bioinformatics phosphorylation predictor, predicted PKM2 might be directly phosphorylated by NEK2 at Thr524. However, the specific phosphorylated sites were not validated in our study.

Having demonstrated that NEK2 could directly interact with PKM2 and modulate the phosphorylation level and protein stability of PKM2, we further explored if NEK2 could influence the cell proliferation and glycolysis in DLBCL *via* PKM2. Functional assay results revealed that overexpression of NEK2 enhanced proliferation and glycolysis of DLBCL cells, and these promoting effects were abrogated by PKM2 inhibitor. These experimental data, therefore, suggest that NEK2 exerts its pro-cancer effect through PKM2 in DLBCL. Lastly, we further constructed a xenograft tumor model to confirm the function of NEK2 *in vivo*. The result shows that decreased NEK2 levels inhibit tumor growth of DLBCL cells *in vivo*.

**Figure 8:**
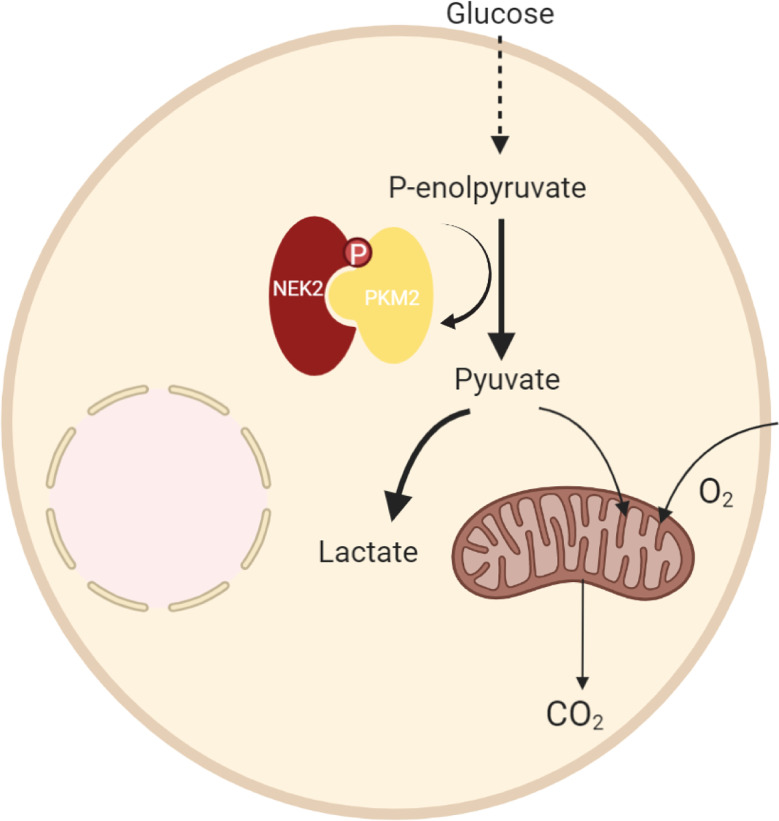
A proposed model to illustrate the regulation of DLBCL by NEK2 through PKM2.

In summary, we demonstrated that NEK2 was highly expressed in DLBCL, and overexpression of NEK2 predicted a worse prognosis of DLBCL patients. Functionally, NEK2 contributed to DLBCL cell proliferation *via* inducing aerobic glycolysis. In regard to mechanism, as the proposed model shows (**Figure 8**), we firstly reported the NEK2 was a new binding partner to PKM2, which is a rate-limiting glycolytic enzyme. Besides, NEK2 regulates PKM2 abundance *via* phosphorylation. Moreover, NEK2 promotes proliferation and glycolysis through PKM2. Taken together, our present study uncovered NEK2 facilitates cell proliferation and glycolysis by regulating PKM2 level through phosphorylation in diffuse large B-cell lymphoma. Our research helps to refine studies about crosstalk between NEK2 and PKM2. These data provided a novel idea for targeted therapy to DLBCL patients, which may significantly improve DLBCL patients’ treatment outcomes.

## Data Availability Statement

The original contributions presented in the study are included in the article/**Supplementary Material**. Further inquiries can be directed to the corresponding authors.

## Ethics Statement

The studies involving human participants were reviewed and approved by Ethics Committee of Zhejiang University. The patients/participants provided their written informed consent to participate in this study. The animal study was reviewed and approved by Animal Ethics Review Committees of Zhejiang University.

## Author Contributions

The LZ conceived and designed the experiments. LZ performed the experiments. LZ and YG collected and analyzed the data. LD, JZhao, JZhang, ZM, ZW, WZ, and RZ provided crucial suggestions, revised the paper and approved the final version. WZ and RZ supervised the study. All authors contributed to the article and approved the submitted version.

## Funding

This work was supported by the grant from the Nature Science Foundation of Zhejiang Province (Nos.352 LQ20H160025 and Nos. Y17H160070).

## Conflict of Interest

The authors declare that the research was conducted in the absence of any commercial or financial relationships that could be construed as a potential conflict of interest.
