# Supplementary material for: NEK2 Promotes Cell Proliferation and Glycolysis by Regulating PKM2 Abundance via Phosphorylation in Diffuse Large B-Cell Lymphoma
Source: Front Oncol. 2021 Jun 8;11:677763. doi: 10.3389/fonc.2021.677763 (PMC8217770; doi:10.3389/fonc.2021.677763)
Supplement: Supplementary file 1 [file DataSheet_1.zip › Supplemenary Figures.DOCX]

Supplementary Material

# Supplementary Data

GEO datasets analyzed in the article obtained from GEO (http://www.ncbi.nlm.nih.gov/geo/).

# Supplementary Figures and Tables

## Supplementary Figures


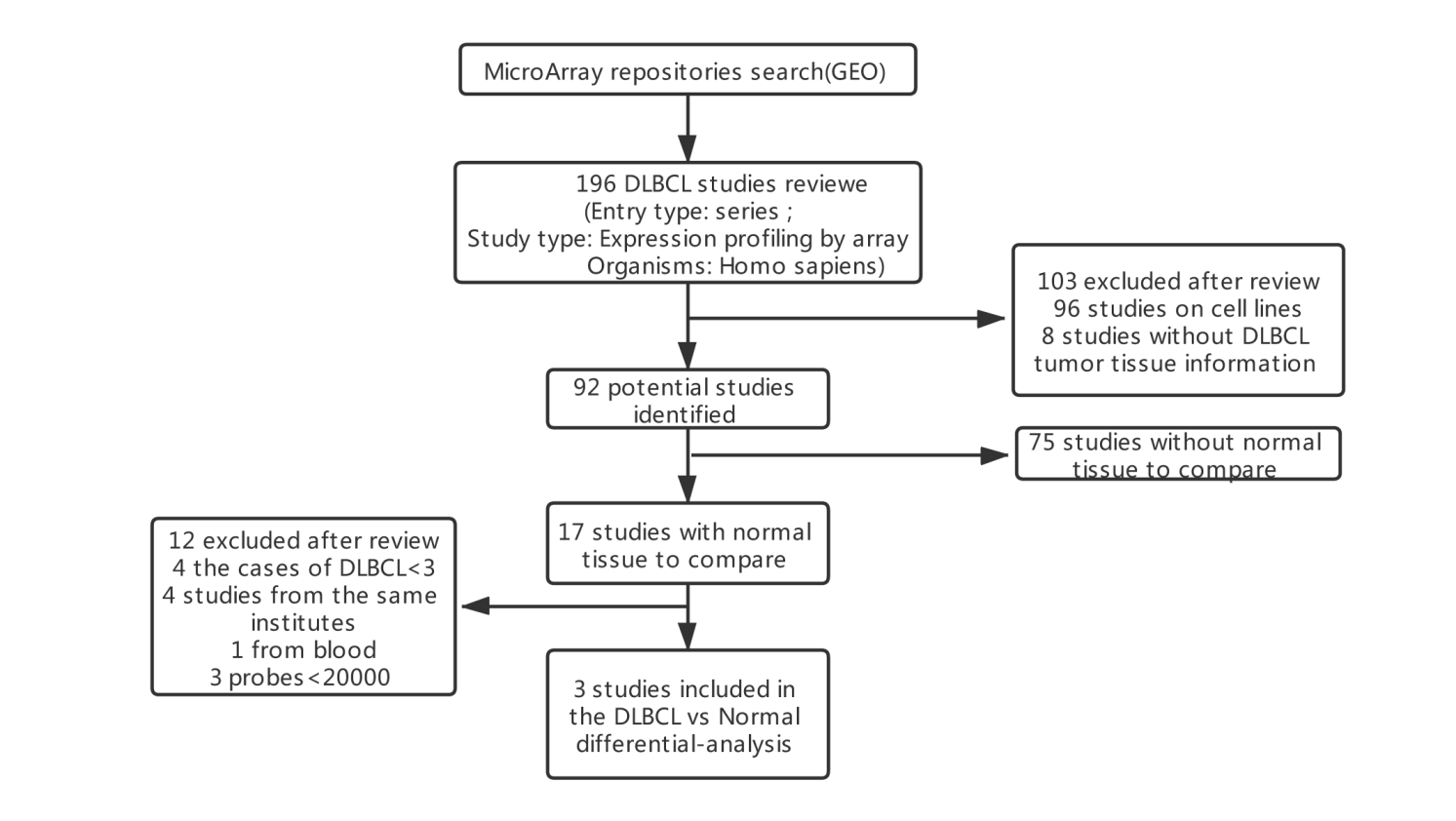


**Supplementary Figure 1 Flow diagram of dataset collection.**

**
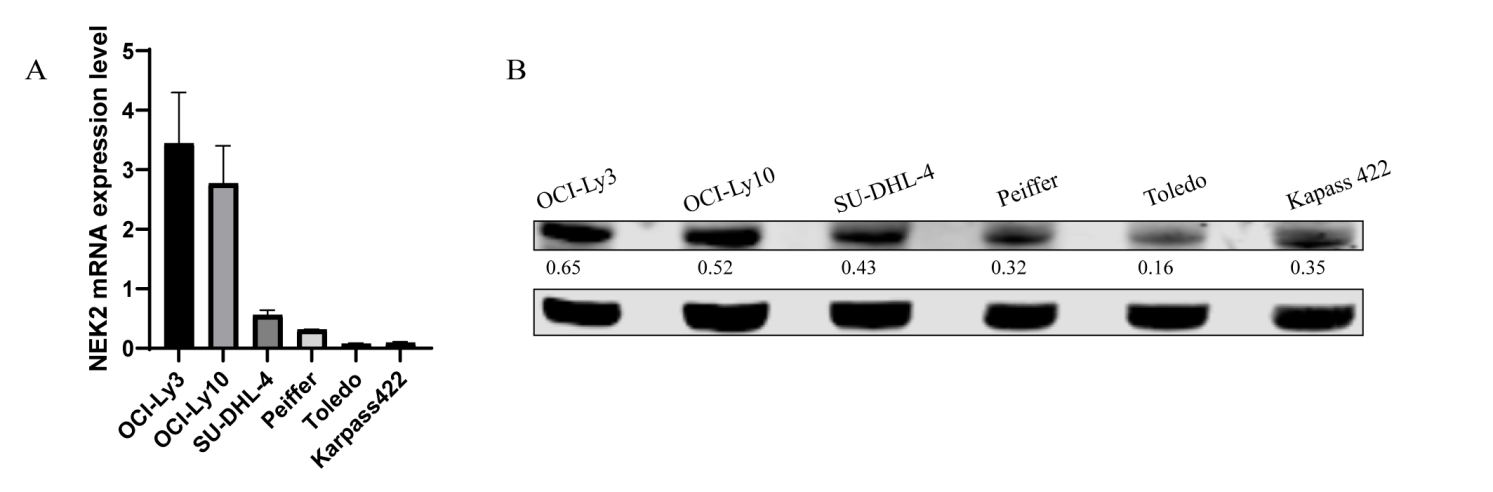
**

**Supplementary Figure 2 NEK2 expression in DLBCL cell lines.** (A) Bar graph shows basal NEK2 mRNA expression level of DLBCL cell lines by qPCR; (B) NEK2 protein expression level of DLBCL cell lines by Western blot analysis.


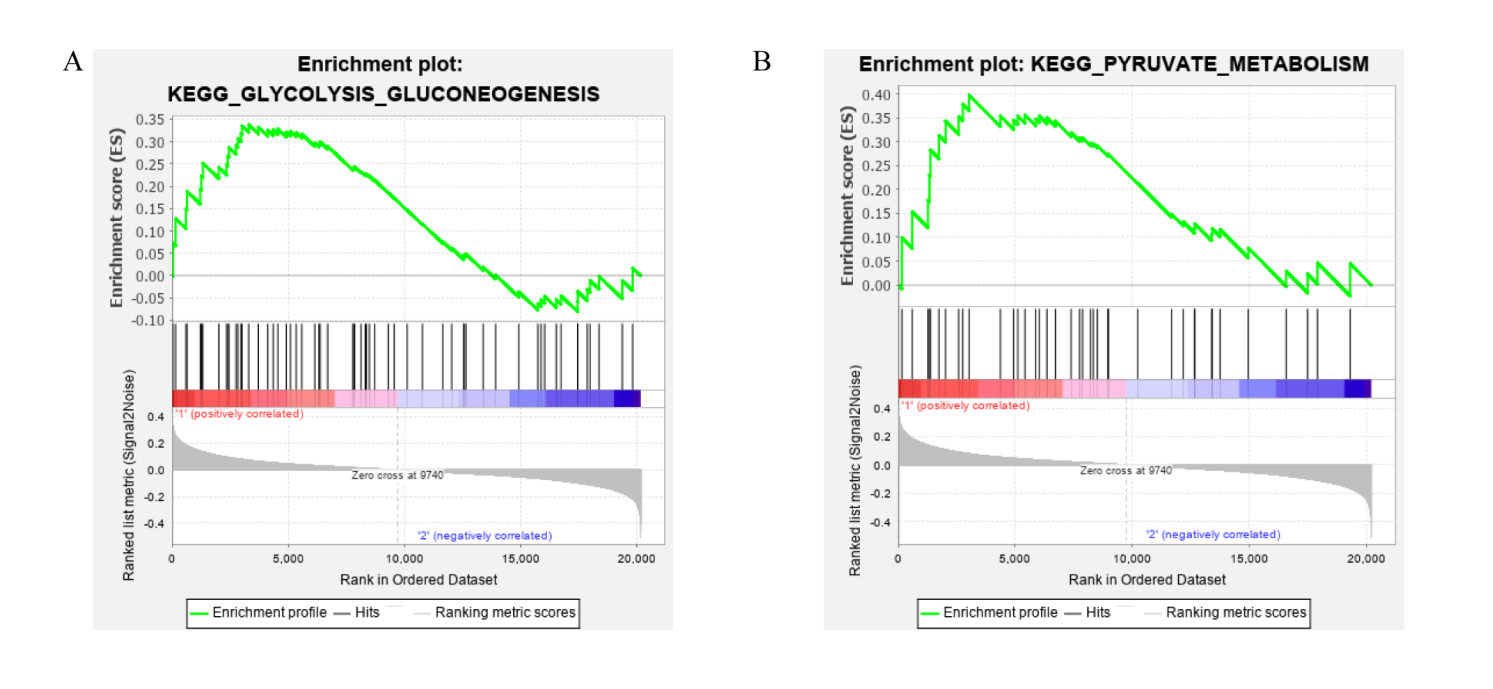


**Supplementary Figure 3 Gene set enrichment analysis in DLBCL.** GSEA results indicating NEK2 might participate the pathways relating to glucose energy metabolism: (A) glycolysis gluconeogenesis; (B) Pyruvate metabolism.

**
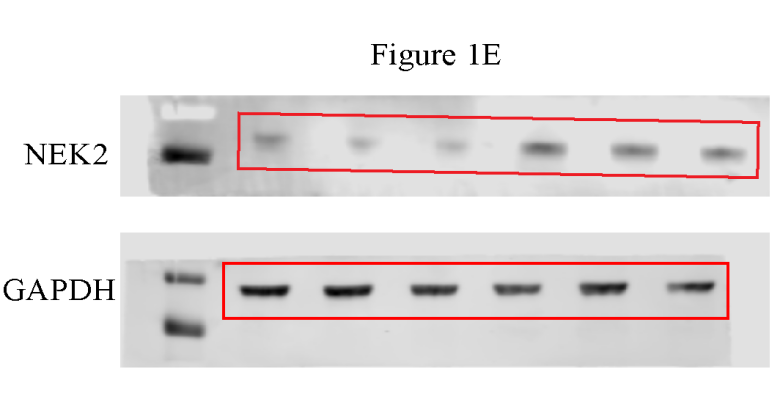
**

**
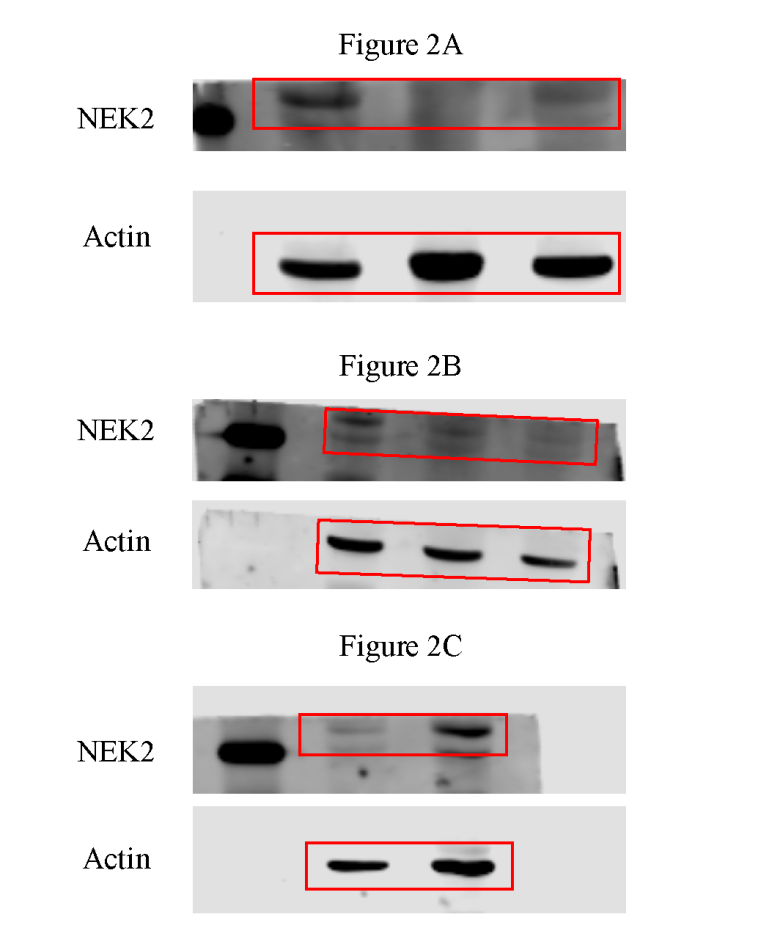
**


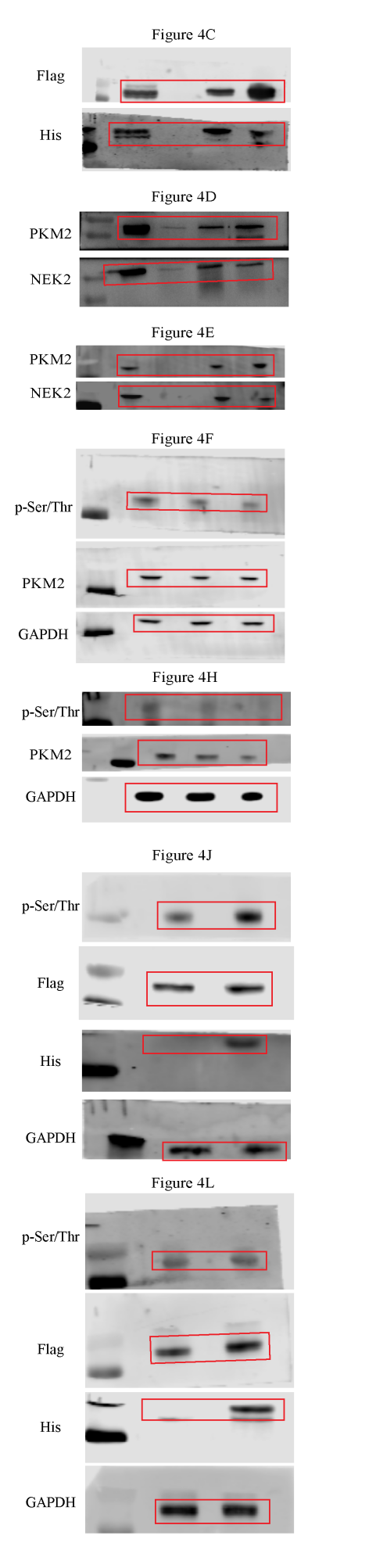
**
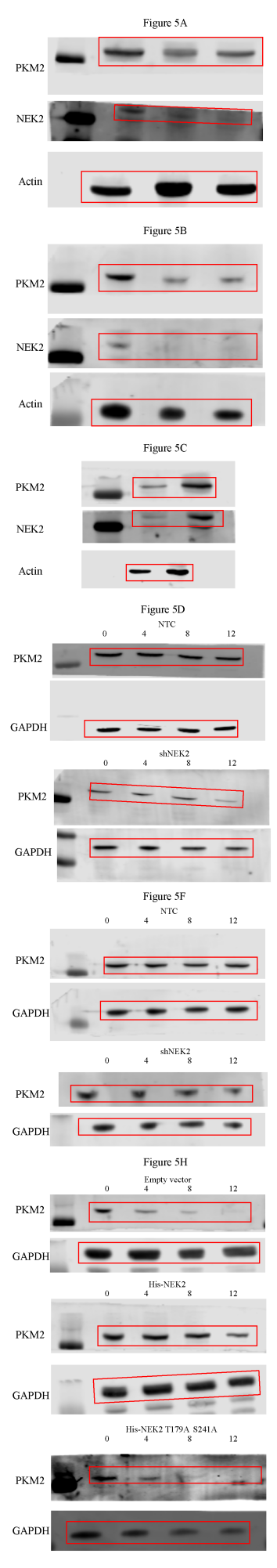
**

**Supplementary Figure 4 Original images of western blot.**

**
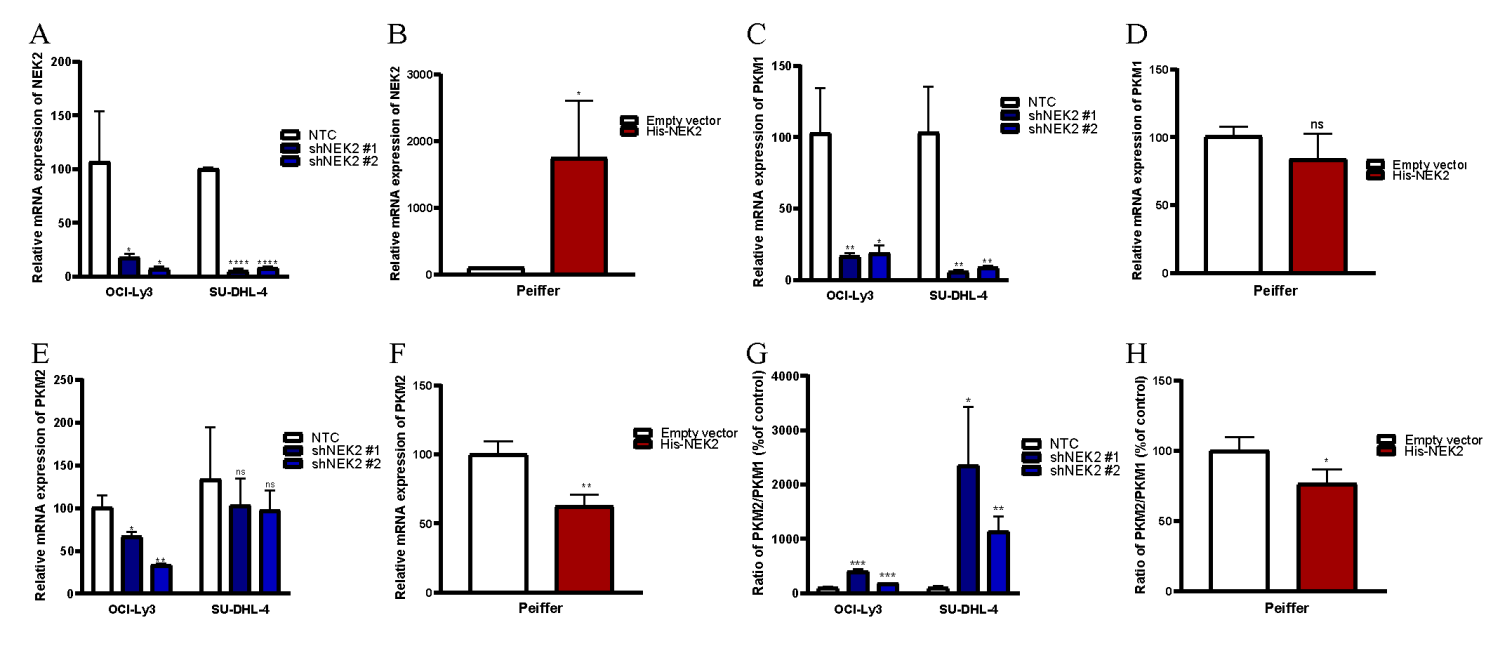
**

**Supplementary Figure 5 The relationship between NEK2 and the ratio of PKM2/PKM1.** (A-H) Real-time PCR analyses of the ratio of PKM2/PKM1 in NEK2 knockdown OCI-Ly3 and SU-DHL-4 cells and NEK3 overexpression Peiffer cells. Results of real-time PCR were normalized against Actin and presented means ± SD of triplicate determinations from an experiment representative of three, *p < 0.05.

## Supplementary Tables

**Supplementary Table1 The primers used for qRT-PCR.**

**Supplementary Table 2 Full-length human NEK2 cDNA with a hexahistidine (6×His) tag.**

**Supplementary Table 3 Full-length human NEK2 cDNA with Threonine 175 to alanine mutation and Serine 241 to alanine mutation as well as a hexahistidine (6×His) tag.**

**Supplementary Table 4 Clinical information of datasets for differential gene expression analysis.**
