# Supplementary material for: NEK2 Promotes Cell Proliferation and Glycolysis by Regulating PKM2 Abundance via Phosphorylation in Diffuse Large B-Cell Lymphoma
Source: Front Oncol. 2021 Jun 8;11:677763. doi: 10.3389/fonc.2021.677763 (PMC8217770; doi:10.3389/fonc.2021.677763)
Supplement: Supplementary file 1 [file DataSheet_1.zip › Supplemenary Table 1.DOCX]

Supplementary Table1 The primers used for qRT-PCR.

| Gene | Primer (5' - 3') |
| --- | --- |
| NEK2 F | TCTGGTCATTGGGCTGCTTG |
| NEK2 R | TTCTTTGCTCGTCTGCAACC |
| PKM2 F | ATTATTTGAGGAACTCCGCCGCCT |
| PKM2 R | ATTCCGGGTCACAGCAATGATGG |
| Actin F | CTCGCCTTTGCCGATCC |
| Actin R | TCTCCATGTCGTCCCAGTTG |

F: forward; R: reverse.
