# Supplementary material for: NEK2 Promotes Cell Proliferation and Glycolysis by Regulating PKM2 Abundance via Phosphorylation in Diffuse Large B-Cell Lymphoma
Source: Front Oncol. 2021 Jun 8;11:677763. doi: 10.3389/fonc.2021.677763 (PMC8217770; doi:10.3389/fonc.2021.677763)
Supplement: Supplementary file 1 [file DataSheet_1.zip › Supplemenary Table 2.DOCX]

Supplementary Table 2 Full-length human NEK2 cDNA with a hexahistidine (6×His) tag.

| Gene | Sequence (5'-3') |
| --- | --- |
| His-NEK2 | 5'-ATGCCTTCCCGGGCTGAGGACTATGAAGTGTTGTACACCATTGGCACAGGCTCCTACGGCCGCTGCCAGAAGATCCGGAGGAAGAGTGATGGCAAGATATTAGTTTGGAAAGAACTTGACTATGGCTCCATGACAGAAGCTGAGAAACAGATGCTTGTTTCTGAAGTGAATTTGCTTCGTGAACTGAAACATCCAAACATCGTTCGTTACTATGATCGGATTATTGACCGGACCAATACAACACTGTACATTGTAATGGAATATTGTGAAGGAGGGGATCTGGCTAGTGTAATTACAAAGGGAACCAAGGAAAGGCAATACTTAGATGAAGAGTTTGTTCTTCGAGTGAT  GACTCAGTTGACTCTGGCCCTGAAGGAATGCCACAGACGAAGTGATGGTGGTCATACCGTATTGCATCGGGATCTGAAACCAGCCAATGTTTTCCTGGATGGCAAGCAAAACGTCAAGCTTGGAGACTTTGGGCTAGCTAGAATATTAAACCATGACACGAGTTTTGCAAAAACATTTGTTGGCACACCTTATTACATGTCTCCTGAACAAATGAATCGCATGTCCTACAATGAGAAATCAGATATCTGGTCATTGGGCTGCTTGCTGTATGAGTTATGTGCATTAATGCCTCCATTTACAGCTTTTAGCCAGAAAGAACTCGCTGGGAAAATCAGAGAAGGCAAATTCA  GGCGAATTCCATACCGTTACTCTGATGAATTGAATGAAATTATTACGAGGATGTTAAACTTAAAGGATTACCATCGACCTTCTGTTGAAGAAATTCTTGAGAACCCTTTAATAGCAGATTTGGTTGCAGACGAGCAAAGAAGAAATCTTGAGAGAAGAGGGCGACAATTAGGAGAGCCAGAAAAATCGCAGGATTCCAGCCCTGTATTGAGTGAGCTGAAACTGAAGGAAATTCAGTTACAGGAGCGAGAGCGAGCTCTCAAAGCAAGAGAAGAAAGATTGGAGCAGAAAGAACAGGAGCTTTGTGTTCGTGAGAGACTAGCAGAGGACAAACTGGCTAGAGCAGAAAATCTGTTGAAGAACTACAGCTTGCTAAAGGAACGGAAGTTCCTGTCTCTGGCAAGTAATCCAGAACTTCTTAATCTTCCATCCTCAGTAATTAAGAAGAAAGTTCATTTCAGTGGGGAAAGTAAAGAGAACATCATGAGGAGTGAGAATTCTGAGAGTCAGCTCACATCTAAGTCCAAGTGCAAGGACCTGAAGAAAAGGCTTCACGCTGCCCAGCTGCGGGCTCAAGCCCTGTCAGATATTGAGAAAAATTACCAACTGAAA AGCAGACAGATCCTGGGCATGCGCCATCATCACCATCACCATTAG -3' |
