# Supplementary material for: NEK2 Promotes Cell Proliferation and Glycolysis by Regulating PKM2 Abundance via Phosphorylation in Diffuse Large B-Cell Lymphoma
Source: Front Oncol. 2021 Jun 8;11:677763. doi: 10.3389/fonc.2021.677763 (PMC8217770; doi:10.3389/fonc.2021.677763)
Supplement: Supplementary file 1 [file DataSheet_1.zip › Supplemenary Table 3.DOCX]

Supplementary Table 3 Full-length human NEK2 cDNA with Threonine 175 to alanine mutation and Serine 241 to alanine mutation as well as a hexahistidine (6×His) tag.

| Gene | Sequence (5'-3') | |
| --- | --- | --- |
| His-NEK2 T175A S241A | | 5'-  GCCACC ATGCCTTCCCGGGCTGAGGACTATGAAGTGTTGTACACCATTGGCACAGGCTCCTACGGCCGCTGCCAGA  AGATCCGGAGGAAGAGTGATGGCAAGATATTAGTTTGGAAAGAACTTGACTATGGCTCCATGACAGAAGC  TGAGAAACAGATGCTTGTTTCTGAAGTGAATTTGCTTCGTGAACTGAAACATCCAAACATCGTTCGTTAC  TATGATCGGATTATTGACCGGACCAATACAACACTGTACATTGTAATGGAATATTGTGAAGGAGGGGATC  TGGCTAGTGTAATTACAAAGGGAACCAAGGAAAGGCAATACTTAGATGAAGAGTTTGTTCTTCGAGTGAT  GACTCAGTTGACTCTGGCCCTGAAGGAATGCCACAGACGAAGTGATGGTGGTCATACCGTATTGCATCGG  GATCTGAAACCAGCCAATGTTTTCCTGGATGGCAAGCAAAACGTCAAGCTTGGAGACTTTGGGCTAGCTA  GAATATTAAACCATGACACGAGTTTTGCAAAAACATTTGTTGGCGCCCCTTATTACATGTCTCCTGAACA  AATGAATCGCATGTCCTACAATGAGAAATCAGATATCTGGTCATTGGGCTGCTTGCTGTATGAGTTATGT  GCATTAATGCCTCCATTTACAGCTTTTAGCCAGAAAGAACTCGCTGGGAAAATCAGAGAAGGCAAATTCA  GGCGAATTCCATACCGTTACGCCGATGAATTGAATGAAATTATTACGAGGATGTTAAACTTAAAGGATTA  CCATCGACCTTCTGTTGAAGAAATTCTTGAGAACCCTTTAATAGCAGATTTGGTTGCAGACGAGCAAAGA  AGAAATCTTGAGAGAAGAGGGCGACAATTAGGAGAGCCAGAAAAATCGCAGGATTCCAGCCCTGTATTGA  GTGAGCTGAAACTGAAGGAAATTCAGTTACAGGAGCGAGAGCGAGCTCTCAAAGCAAGAGAAGAAAGATT  GGAGCAGAAAGAACAGGAGCTTTGTGTTCGTGAGAGACTAGCAGAGGACAAACTGGCTAGAGCAGAAAAT  CTGTTGAAGAACTACAGCTTGCTAAAGGAACGGAAGTTCCTGTCTCTGGCAAGTAATCCAGAACTTCTTA  ATCTTCCATCCTCAGTAATTAAGAAGAAAGTTCATTTCAGTGGGGAAAGTAAAGAGAACATCATGAGGAG  TGAGAATTCTGAGAGTCAGCTCACATCTAAGTCCAAGTGCAAGGACCTGAAGAAAAGGCTTCACGCTGCC  CAGCTGCGGGCTCAAGCCCTGTCAGATATTGAGAAAAATTACCAACTGAAAAGCAGACAGATCCTGGGCA  TGCGCCATCATCACCATCACCATTAG -3' |
