# Supplementary material for: NEK2 Promotes Cell Proliferation and Glycolysis by Regulating PKM2 Abundance via Phosphorylation in Diffuse Large B-Cell Lymphoma
Source: Front Oncol. 2021 Jun 8;11:677763. doi: 10.3389/fonc.2021.677763 (PMC8217770; doi:10.3389/fonc.2021.677763)
Supplement: Supplementary file 1 [file DataSheet_1.zip › Supplemenary Table 4.DOCX]

Supplementary Table 4 Clinical information of datasets for differential gene expression analysis.

| Study | Year | Patient sorce | GEO | Platform | Raw data type | Sample DLBCL | Type  normal |
| --- | --- | --- | --- | --- | --- | --- | --- |
| Victor Segura | 2012 | Spain | GSE25638 | GPL570 | TAR | 26 | 13 |
| Siegfried Janz | 2013 | USA | GSE44337 | GPL570 | TAR | 9 | 3 |
| Julie Støve | 2015 | Denmark | GSE56315 | GPL570 | TAR | 55 | 33 |
